# Long-term health consequences among individuals with SARS-CoV-2 infection compared to individuals without infection: results of the population-based cohort study CoMoLo Follow-up

**DOI:** 10.1186/s12889-023-16524-8

**Published:** 2023-08-21

**Authors:** Christin Heidemann, Giselle Sarganas, Yong Du, Beate Gaertner, Christina Poethko-Müller, Caroline Cohrdes, Sein Schmidt, Martin Schlaud, Christa Scheidt-Nave

**Affiliations:** 1 Department of Epidemiology and Health Monitoring, Robert Koch Institute, Berlin, Germany; 2 Clinical Study Center, Berlin Institute of Health at Charité – Universitätsmedizin Berlin, Berlin, Germany

**Keywords:** SARS-CoV-2, COVID-19, Post COVID, Long COVID, Follow-up, Symptoms, Subjective health, Health-related quality of life, Patient-reported outcomes, Fatigue

## Abstract

**Background:**

Most of the previous studies on health sequelae of COVID-19 are uncontrolled cohorts and include a relatively short follow-up. This population-based multi-center cohort study examined health consequences among individuals about 1 to 1.5 years after SARS-CoV-2 infection compared with non-infected.

**Methods:**

The study population consisted of adults (≥ 18 years) from four municipalities particularly affected by the COVID-19 pandemic in the year 2020 who completed a detailed follow-up questionnaire on health-related topics. Exposure was the SARS-CoV-2 infection status (based on IgG antibodies, PCR test, or physician-diagnosis of COVID-19) at baseline (May to December 2020). Outcomes assessed at follow-up (October 2021 to January 2022; mean: 452 days) included recurrent or persistent health complaints, incident diseases, health-related quality of life (PROMIS-29), subjective health, and subjective memory impairment. Logistic and linear regression models were adjusted for baseline sociodemographic and lifestyle characteristics (age, sex, municipality, education, smoking, body mass index), pre-existing health conditions (chronic disease/health problem, health-related activity limitation, depressive/anxiety disorder), and follow-up time.

**Results:**

Among 4817 participants, 350 had a SARS-CoV-2 infection at baseline and 4467 had no infection at baseline or during follow-up. Those with an infection statistically significantly more often reported 7 out of 18 recurrent or persistent health complaints at follow-up: smell/taste disorders (12.8% vs. 3.4%, OR 4.11), shortness of breath (23.0% vs. 9.5%, 3.46), pain when breathing (4.7% vs. 1.9%, 2.36), fatigue (36.9% vs. 26.1%, 1.76), weakness in legs (12.8% vs. 7.8%, 1.93), myalgia/joint pain (21.9% vs. 15.1%, 1.53) and cough (30.8% vs. 24.8%, 1.34) and 3 out of 6 groups of incident diseases: liver/kidney (2.7% vs. 0.9%, 3.70), lung (3.2% vs. 1.1%, 3.50) and cardiovascular/metabolic (6.5% vs. 4.0%, 1.68) diseases. Those with an infection were significantly more likely to report poor subjective health (19.3% vs. 13.0%, 1.91), memory impairment (25.7% vs. 14.3%, 2.27), and worse mean scores on fatigue and physical function domains of PROMIS-29 than non-infected.

**Conclusion:**

Even after more than one year, individuals with SARS-CoV-2 infection showed an increased risk of various health complaints, functional limitations, and worse subjective well-being, pointing toward profound health consequences of SARS-CoV-2 infection relevant for public health.

**Supplementary Information:**

The online version contains supplementary material available at 10.1186/s12889-023-16524-8.

## Background

As observed with other viral infections, patients who recovered from SARS-CoV-2 infection have experienced a wide variety of systemic and organ-specific health conditions, which can persist or newly occur beyond the acute phase of the infection [1–3]. The diverse clinical picture has hampered the development of a clinical case definition and hence the early diagnosis and treatment of patients with long-term sequelae of SARS-CoV-2 infection [1, 2]. Patients themselves coined the term long COVID [4]. In order to aid clinical decision making and streamline investigations in epidemiological and clinical studies, the World Health Organization proposed a preliminary clinical case definition in October 2021 defining post COVID-19 condition as a condition characterized by symptoms usually occurring three months from the onset of an acute SARS-CoV-2 infection that last for at least two months and cannot be explained by an alternative diagnosis [5]. National clinical guidelines on the management of patients with post-acute sequelae of SARS-CoV-2 infection (PASC) usually apply the terms post COVID-19 condition or post COVID-19 syndrome to summarize otherwise unexplained symptoms or health conditions that are still present three months and longer after SARS-CoV-2 infection; the term long COVID is used to describe clinical signs that are present after a defined four week period of acute COVID-19 [6, 7].

Commonly described long COVID symptoms include fatigue, shortness of breath and cognitive dysfunction among others [1, 5]. Moreover, long COVID has been associated with limitations on daily living activities and reduced quality of life [8, 9] as well as an increased risk of newly diagnosed common chronic diseases, including chronic respiratory, cardiovascular, neuropsychiatric, and autoimmune conditions [2, 10, 11]. However, few population-based studies have so far comprehensively examined the long-term health consequences among individuals with SARS-CoV-2 infection compared to those who were not infected, which is crucial for validly identifying long COVID [12, 13].

In Germany, the regional cumulative incidence of SARS-CoV-2 infection varied extremely at the beginning of the pandemic. In four of the most affected municipalities, the study CORONA-MONITORING local (CoMoLo) was conducted to obtain the population-based seroprevalence of COVID-19 infection at the early stage of the pandemic. As part of a 1-year-follow-up of the study population, the present study examined the potential long-term health consequences of SARS-CoV-2 infection, comparing individuals with and without SARS-CoV-2 infection with respect to predefined health outcomes.

## Methods

### The study CORONA-MONITORING lokal (CoMoLo) and its follow-up

The CoMoLo baseline study was a population-based study to investigate the seroprevalence of SARS-CoV-2 antibodies in four selected municipalities in Germany that were particularly affected by the COVID-19 pandemic in the year 2020 (i.e. with a reported cumulative SARS-CoV-2 incidence of more than 500 cases per 100,000 inhabitants before the start of the study). At baseline, random samples of adults (aged ≥ 18 years, *n* = 8999) from the local population registration offices were asked to visit a temporary study center to collect a blood sample and an oropharyngeal swab and to complete a short written questionnaire. About one to two weeks later, a detailed web-based or telephone-based questionnaire was completed [14, 15]. Study periods were May/June 2020 for Kupferzell (response proportion 63%), June/July 2020 for Bad Feilnbach (response proportion 59%), September 2020 for Straubing (response proportion 30%) and November/December 2020 for Berlin-Mitte (response proportion 29%) [16, 17]. Further details of the study have been described previously [14–17].

In 2021/2022, a follow-up study was conducted with participants who had given consent for re-contact (*n* = 8372) to investigate, among other aspects, (a) long-term health-consequences in association with infection status for all participants and (b) seroprevalence in comparison to baseline for participants in one municipality (Straubing). To achieve these aims, participants from all municipalities were asked between October 2021 and January 2022 to complete a detailed web-based or telephone-based questionnaire on health-related topics (response proportion among those eligible for follow-up-invitation 65%). All participants of Straubing were also invited for on-site follow-up blood sampling.

### Study population

**Fig. 1:**
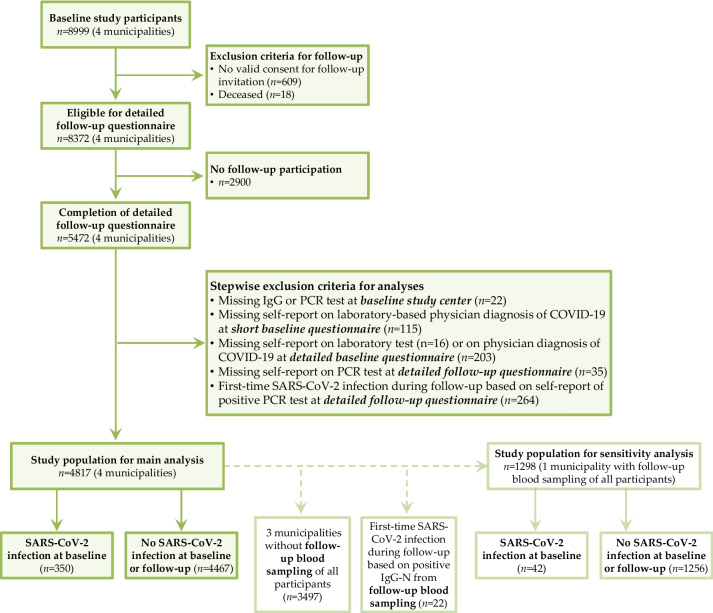
Flow chart with stepwise exclusion criteria applied to define the study population

For the present data analysis on long-term health consequences of SARS-CoV-2 infections, the study population consisted of baseline study participants from the four municipalities who completed the detailed follow-up questionnaire on health-related topics in 2021/2022 (*n* = 5472). The flow chart (Fig. 1) reflects the numbers of participants who were stepwise excluded according to defined criteria (i.e. missing information to define infection status at baseline and follow-up (*n* = 391) as well as first-time SARS-CoV-2 infection during follow-up (*n* = 264)) and the final number of participants included in the present analysis (*n* = 4817). In addition, the numbers of the study population differentiated by infection status are given.

For one municipality (Straubing), blood samples were taken from all participants at follow-up. Thus, in addition to the information from the follow-up questionnaire, blood sample results could be considered for an expanded definition of infection status at follow-up for a sensitivity analysis. The final number of participants included in the sensitivity analysis (*n* = 1298) is also shown in the flow chart.

### Exposure assessment of SARS-CoV-2 infection status

For all four municipalities, the same criteria were applied to define infection status. The applied criteria were dependent by the availability of PCR tests and vaccines. At baseline in the year 2020, PCR tests were not widely feasible and vaccines against SARS-CoV-2 were not yet available. At the time of follow-up in the 2021/2022 both were widely available.

Participants with SARS-CoV-2 infection at baseline were defined based on (a) detectable IgG antibodies against the virus’ S1 antigen from venous blood specimens (Anti-SARS-CoV-2-QuantiVac-ELISA (IgG), Euroimmun, Lübeck, Germany) (*n* = 289) or a positive PCR test result based on an oropharyngeal swab (SARS-CoV-2 RT-PCR testing targeting the E gene and the orf1ab region of SARS-CoV-2) (*n* = 6) taken at the study center [14, 15] or (b) a self-reported history of laboratory-based physician diagnosis of COVID-19 (*n* = 29) in the short written questionnaire completed at the study center or (c) a self-reported positive laboratory test (*n* = 15) or a physician diagnosis of COVID-19 (*n* = 11) in the detailed questionnaire one to two weeks later.

Participants without a SARS-CoV-2 infection were defined as participants (a) without an infection at baseline (as described above) and (b) without a first-time infection during follow-up. First-time infection during follow-up was defined based on a self-reported history of a positive PCR test in the detailed follow-up questionnaire.

For one municipality (Straubing), venous blood samples were taken from all participants at follow-up, so that in a sensitivity analysis of the present study, detectable IgG antibodies against the virus’ N-antigen (Elecsys Anti-SARS-CoV-2, Roche, Basel, Switzerland) were additionally considered for defining a first-time SARS-CoV-2 infection during follow-up.

### Outcome assessment of health consequences at follow-up

All health consequences at follow-up were assessed in the detailed follow-up questionnaire. Recurrent or persistent health complaints since baseline participation at the study center were assessed based on a list of 18 complaints (in the order as listed in Table 2). The total number of health complaints was categorized into 0, 1–2, 3–4 and ≥ 5. In case of indicating a complaint, participants were further asked whether the complaint still exists until today. Incident physician-diagnosed diseases since baseline participation at the study center were assessed based on a list of eight disease groups, i.e. comprising cardiovascular disease (e.g. myocarditis, heart failure/cardiac insufficiency, heart attack, stroke, venous thrombosis), lung disease (e.g. pneumonia, pulmonary embolism, pulmonary fibrosis), gastrointestinal disease, liver disease, kidney disease, neurological disease (e.g. Parkinson's disease, neuropathies), mental illness (e.g. depression, anxiety disorder), and metabolic disease (e.g. diabetes mellitus). Multiple answers regarding recurrent or persistent health complaints and incident diseases were possible.

Current subjective health was measured by the question “What is your state of health in general?” (response options: very good, good, fair, bad, very bad). Subjective memory impairment was defined as self-reported memory worsening with associated worries [18]. Different aspects of current health-related quality of life (i. e. physical, mental, and social aspects) were assessed by the German-language version of the Patient-Reported Outcomes Measurement Information System (PROMIS)-29 Profile v2.1 [19]. The PROMIS-29 Profile v2.1 measures seven domains: physical function, fatigue, sleep disturbance, pain interference (as domains of physical well-being), anxiety, depression (as domains of mental health), and ability to participate in social roles and activities (as domain of social well-being) with each including four items (each scored on a 5-point scale) and, additionally, a single item on pain intensity (scored on a 10-point scale). Higher scores reflect more of the domain’s concept being measured, i. e. better physical function and ability to participate in social roles and activities, but worse anxiety, depression, fatigue, sleep disturbance, pain interference and pain intensity [20]. Raw domain scores were obtained and converted into standardized t-scores with response pattern scoring following the PROMIS scoring manual [21].

### Assessment of baseline characteristics (covariables)

Age, sex, school qualification and smoking status were assessed in the short-written baseline questionnaire. All other information was self-reported in the detailed baseline questionnaire. Educational status (lower, medium, high) was defined by information on school qualification (4 categories) and on vocational qualification (14 categories) using the 2011 version of the International Standard Classification of Education (ISCED) [22]. Smoking status (current, ex-, never smoking) was assessed by the information of daily, occasionally, no more, or never smoking. Body mass index (BMI) was calculated from self-reported body height and body weight (without clothes and shoes) and a BMI ≥ 30 kg/m^2^ was considered as obesity (yes/ no). Presence of chronic illness, i.e. of any chronic disease or long-term health problem lasting for at least six months (yes/no), and presence of any severely or moderately health-related limitation in usual everyday activities lasting for at least six months (yes/no) were recorded, which both are established central indicators of the general health status [23]. Presence of depression or anxiety disorders in the past two weeks was defined as an indicator of mental health by the Patients Health Questionnaire-4 (PHQ-4), which consists of a 2-item depression scale (PHQ-2) and a 2-item anxiety scale (GAD-2), and was assumed in case of a sum score of at least six points (possible score range: 0–12 points) [24].

### Statistical analysis

All statistical analyses were performed using Stata (version 17.0, StataCorp, College Station, TX, USA). Proportions and their 95% confidence intervals (95% CI) for categorical variables as well as means and their 95% CI for continuous variables, along with numbers of observations, were reported for description of possible health consequences of SARS-CoV-2 infection at follow-up. For logistic and linear regression analyses, suspected potential confounding variables for the association between SARS-CoV-2 infection and long-term health consequences were included as covariables. The basic models were adjusted for the baseline information on sociodemographic characteristics (age, sex, municipality, and educational level) and the fully-adjusted models were additionally adjusted for baseline information on health-related characteristics (obesity, smoking status, chronic disease/health problem, health-related activity limitations, depressive/anxiety disorders) and follow-up time (days). As results between basic and fully-adjusted models differed only marginally, we present only results from the fully-adjusted models. Since the impact of a risk factor depends on both its frequency in the population and the strength of its association with the health outcome of interest, we also examined population attributable risks (PAR). PAR were derived by an equation postulated by Miettinen, taking the relative frequency of infections among participants with the respective health consequence and the respective odds ratio from the fully-adjusted model into consideration [25].

Proportions of missing information in baseline variables ranged from 0.04% for educational level, 0.4% for chronic disease/health problem and health-related activity limitation, 1.4% for depressive/anxiety disorders to 1.9% for smoking status. Persons with missing information were excluded from the descriptive analysis of baseline characteristics (as described in footnote of Table 1). To handle missing information for regression analyses, multiple imputation of missing baseline information using multiple regression analyses with the fully conditional specification (chained equations) approach was performed, assuming missing at random [26]. A total of 15 imputed datasets were created. Imputed data sets were analyzed combined to obtain odds ratios (OR) and 95% CI from logistic regression analysis or β coefficients and 95% CI from linear regression analysis, respectively, taking the within-variation and between-variation of imputed data sets into account.

## Results

### Baseline characteristics of the study population

Among the 4817 adults included in the analysis, slightly more than half were female. The mean age was 49.8 years with less than 10% of participants aged 75 years or older (Table 1). Most participants had a high or medium educational background. Half of participants had never smoked, about one third were ex-smokers, and one fifth were current smokers. About 17% of participants were obese. More than one third reported a chronic disease or health problem, and nearly one fifth reported health-related activity limitations. Less than 10% of the study population indicated depressive or anxiety disorders.

**Table 1:** Baseline characteristics of the study population overall and differentiated by SARS-CoV-2 infection status

			**Stratified by SARS-CoV-2 infection status**			
	**Total (*n* = 4817)**		**Infection at baseline (*n* = 350)**		**No infection (*n* = 4467)**	
**Baseline characteristics**	***n***	**% (95% CI) or mean** ± **SD**	***n***	**% (95% CI) or mean** ± **SD**	***n***	**% (95% CI) or mean** ± **SD**
Female sex (%)	2593	53.8 (52.4-55.2)	208	59.4 (54.2–64.5)	2385	53.4 (51.9–54.9)
Age group (%)						
18–49 years	2356	48.9 (47.5–50.3)	167	47.7 (42.5–53.0)	2189	49.0 (47.6–50.5)
50–74 years	2030	42.1 (40.8–43.5)	146	41.7 (36.6–47.0)	1884	42.2 (40.7–43.6)
≥ 75 years	431	8.9 (8.2–9.8)	37	10.6 (7.7–14.3)	394	8.8 (8.0–9.7)
Age in years (mean)	4817	49.8 ± 17.3	350	49.3 ± 18.4	4467	49.8 ± 17.3
Municipality (%)						
Kupferzell	1191	24.7 (23.5–26.0)	151	43.1 (38.0–48.4)	1040	23.3 (22.1–24.5)
Bad Feilnbach	1049	21.8 (20.6–23.0)	95	27.1 (22.7–32.1)	954	21.4 (20.2–22.6)
Straubing	1320	27.4 (26.2–28.7)	42	12.0 (9.0–15.9)	1278	28.6 (27.3–30.0)
Berlin-Mitte	1257	26.1 (24.9–27.4)	62	17.7 (14.0–22.1)	1195	26.8 (25.5–28.1)
Educational level (%)						
Lower	337	7.0 (6.3–7.8)	41	11.7 (8.7–15.5)	296	6.6 (5.9–7.4)
Medium	2170	45.1 (43.7–46.5)	177	50.6 (45.3–55.8)	1993	44.6 (43.2–46.1)
High	2308	47.9 (46.5–49.3)	132	37.7 (32.8–42.9)	2176	48.7 (47.3–50.2)
Smoking status (%)						
Current smoker	948	20.1 (18.9–21.2)	45	13.1 (9.9–17.1)	903	20.6 (19.4–21.8)
Ex-smoker	1431	30.3 (29.0–31.6)	93	27.1 (22.7–32.1)	1338	30.5 (29.2–31.9)
Never smoker	2347	49.7 (48.2–51.1)	205	59.8 (54.5–64.8)	2142	48.9 (47.4–50.4)
Obesity (BMI ≥ 30 kg/m2; %)	802	16.8 (15.7–17.8)	55	16.5 (12.9–20.9)	747	16.8 (15.7–17.9)
BMI (kg/m^2^; mean)	4786	25.9 ± 5.1	333	25.8 ± 4.8	4453	25.9 ± 5.1
Chronic disease or health problem (%)	1682	35.0 (33.7–36.4)	110	32.9 (28.1–38.2)	1572	35.2 (33.8–36.6)
Health-related activity limitations (%)	924	19.3 (18.2–20.4)	53	15.9 (12.3–20.2)	871	19.5 (18.4–20.7)
Depression or anxiety disorders (%)	299	6.3 (5.6–7.0)	17	5.1 (3.2–8.1)	282	6.4 (5.7–7.1)

*BMI* Body mass index

Missings for education level *n* = 2, smoking status *n* = 91, body mass index *n* = 31, chronic disease or health problem *n* = 18, health-related activity limitation *n* = 18, depression/anxiety disorders *n* = 64

A total of 350 participants (7.3% of the study population) had a SARS-CoV-2 infection at baseline. Among these, 6 participants reported to have been hospitalized due to COVID-19 and none was treated in an intensive care unit. A total of 4467 participants had no evidence of SARS-CoV-2 infection at baseline or during follow-up. The two groups differentiated by SARS-CoV-2 infection status did not significantly differ regarding baseline characteristics, including age distribution, the proportion of obesity, and a history of chronic diseases, health-related activity limitations and depressive or anxiety disorders. However, individuals with infection at baseline were more likely to be female, to have a lower educational level, and to be never smokers compared to individuals without SARS-CoV-2 infection.

### Follow-up time

Mean follow-up time, i.e. mean time between baseline study center visit and completion of the detailed follow-up questionnaire, was 452 days (range: 335 to 607 days) for the total study population and varied slightly between participants with infection at baseline (486 days, range: 341 to 601 days) and participants without an infection (449 days, range: 335 to 607 days).

### Self-reported recurrent or persistent health complaints in association with SARS-CoV-2 infection

At follow-up, most frequently reported recurrent or persistent health complaints since baseline participation among both participants with and without baseline SARS-CoV-2 infection included a runny nose, headaches, and fatigue, followed by cough, a sore throat, and sleep disorders (Table 2). After adjusting for potential confounders in multivariable regression analyses, the following complaints were found to be significantly associated with baseline SARS-CoV-2 infection: smell and taste disorders (OR 4.11, 95% CI 2.84–5.96), shortness of breath (3.46, 2.56–4.68), pain when breathing (2.36, 1.32–4.22), fatigue (1.76, 1.38–2.24), weakness in the legs (1.93, 1.32–2.80), myalgia and joint pain (1.53, 1.15–2.04), and cough (1.34, 1.04–1.71).

**Table 2:** Recurrent or persistent health complaints at follow-up comparing participants with and without SARS-CoV-2 infection

Recurrent or persistent health complaints	Infection at baseline (*n* = 350)			No infection (*n* = 4467)		
	***n***	**% (95% CI)**	**Odds ratio (95% CI)**	***n***	**% (95% CI)**	**Odds ratio (95% CI)**
Fever above 38 °C	20	5.8 (3.8–8.9)	0.92 (0.57–1.49)	274	6.3 (5.7–7.1)	reference
Cough	106	30.8 (26.1–35.9)	1.34 (1.04–1.71)	1073	24.8 (23.6–26.1)	reference
Shortness of breath	79	23.0 (18.8–27.7)	3.46 (2.56–4.68)	412	9.5 (8.7–10.4)	reference
Pain when breathing	16	4.7 (2.9–7.5)	2.36 (1.32–4.22)	80	1.9 (1.5–2.3)	reference
Chest pain	23	6.7 (4.5–9.9)	1.34 (0.84–2.12)	224	5.2 (4.6–5.9)	reference
Sore throat	89	25.9 (21.5–30.8)	0.95 (0.73–1.24)	1114	25.8 (24.5–27.1)	reference
Runny nose	132	38.4 (33.4–43.6)	0.98 (0.77–1.24)	1638	37.9 (36.5–39.4)	reference
Smell/taste disorders	44	12.8 (9.6–16.8)	4.11 (2.84–5.96)	147	3.4 (2.9–4.0)	reference
Loss of appetite	19	5.5 (3.5–8.5)	1.36 (0.82–2.26)	185	4.3 (3.7–4.9)	reference
Fatigue	127	36.9 (32.0–42.2)	1.76 (1.38–2.24)	1127	26.1 (24.8–27.4)	reference
Headaches	113	32.9 (28.2–38.1)	0.90 (0.70–1.15)	1485	34.4 (33.0–35.8)	reference
Dizziness	59	17.2 (13.5–21.5)	1.17 (0.86–1.59)	635	14.7 (13.7–15.8)	reference
Nausea/stomach upset	45	13.1 (9.9–17.1)	0.91 (0.65–1.28)	601	13.9 (12.9–15.0)	reference
Hot flushes/chills	37	10.8 (7.9–14.5)	0.80 (0.56–1.16)	516	11.9 (11.0–12.9)	reference
Sleep disorders	76	22.1 (18.0–26.8)	0.94 (0.71–1.24)	1004	23.2 (22.0–24.5)	reference
Myalgia and joint pain	75	21.9 (17.8–26.6)	1.53 (1.15–2.04)	652	15.1 (14.1–16.2)	reference
Numbness/burning/tingling in the feet/legs/hands	28	8.1 (5.7–11.6)	1.18 (0.78–1.84)	299	6.9 (6.2–7.7)	reference
Weakness in the legs	44	12.8 (9.6–16.8)	1.93 (1.32–2.80)	335	7.8 (7.0–8.6)	reference

Odds ratio and 95% confidence interval (95% CI) are derived from logistic regression models adjusted for baseline information on age (years), sex, municipality, educational level (lower, medium, high), obesity (BMI ≥ 30 vs. < 30 kg/m^2^), smoking status (current, ex-, never smoker), chronic disease/health problem (yes vs. no), health-related activity limitation (yes vs. no), depressive/anxiety disorders (yes vs. no), follow-up time (days). Missings for cough, shortness of breath, sore throat, runny nose, loss of appetite, fatigue, dizziness, numbness/burning/tingling in the feet/legs/hands *n* = 149, pain when breathing, headache, nausea/stomach upset, hot flushes/chills, sleep disorders *n* = 150, fever above 38 °C, weakness in the legs *n* = 151, smell/taste disorders *n* = 152, chest pain, myalgia and joint pain* n* = 153

According to estimated PAR, the highest proportions out of these seven health complaints attributable to baseline infection were obtained for smell and taste disorders (17.4%, 95% CI 14.9–19.2%), shortness of breath (11.4%, 9.8–12.7%) and pain when breathing (9.6%, 4.0–12.7%), whereas lower proportions were obtained for weakness in the legs (5.6%, 2.8–7.5%), fatigue (4.4%, 2.8–5.6%), myalgia and joint pain (3.6%, 1.3–5.3%) and cough (2.3%, 0.3–3.7%).

**Fig. 2:**
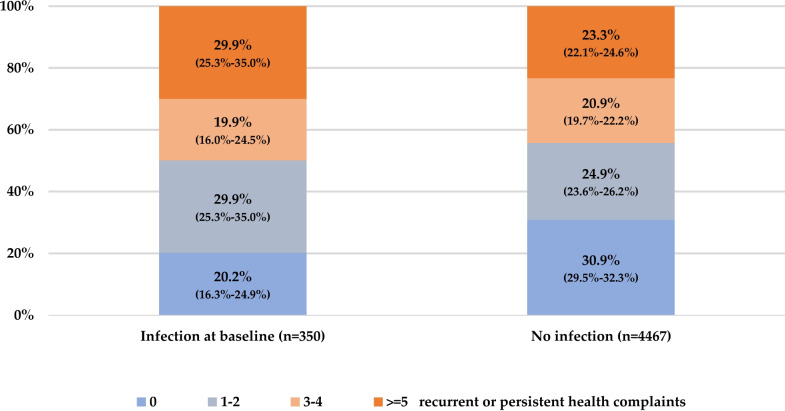
Number of recurrent or persistent health complaints at follow-up comparing participants with and without SARS-CoV-2 infection

Overall, 30.9% of the participants without an infection stated none of the 18 listed recurrent or persistent health complaints compared to 20.2% of those with infection at baseline. Whereas five health complaints or more were more frequent among participants with infection at baseline (29.9%) than among participants without infection (23.3%; Fig. 2). When applying multivariable ordinal regression analysis, SARS-CoV-2 infection was significantly associated with a higher number of recurrent or persistent health complaints (OR 1.43, 95% CI 1.17–1.75).

Results from multivariable regression analyses largely persisted when considering only recurrent or persistent health complaints that were reported to be still present at the date of interview at follow-up (Supplementary table 1). The frequencies of recurrent or persistent health complaints lasting until the date of follow-up were expectedly lower than those reported in Table 2. Still, the absence of all 18 health complaints was less likely among participants with infection at baseline (45.9%) than among participants without infection (60.0%; Supplementary Fig. 1). In multivariable ordinal regression analysis, SARS-CoV-2 infection remained significantly associated with a higher number of health complaints (OR 1.83, 95% CI 1.47–2.28).

Results were also similar, when we restricted the analyses to data from one municipality (Straubing), where participants underwent seroprevalence testing both at baseline and follow-up (Fig. 1). However, the observed group differences and associations between SARS-CoV-2 infection and health complaints were not always statistically significant, probably due to the considerably lower absolute number of participants as only one municipality was considered (Supplementary table 2).

### History of newly diagnosed diseases in association with SARS-CoV-2 infection

Among both groups, participants with and without baseline SARS-CoV-2 infection, cardiovascular and metabolic diseases were the most frequently reported newly diagnosed diseases since baseline participation (Table 3). After adjusting for potential confounders, including available information on health status at baseline, in multivariable regression models, SARS-CoV-2 infection was observed to be significantly associated with newly diagnosed liver or kidney diseases (OR 3.70, 95% CI 1.69–8.08), lung diseases (3.50, 1.73–7.09), and cardiovascular or metabolic diseases (1.68, 1.04–2.71).

**Table 3:** Incident diseases, subjective health, and subjective memory impairment at follow-up comparing participants with and without SARS-CoV-2 infection

	**Infection at baseline (*n* = 350)**			**No infection (*n* = 4467)**		
	***n***	**% (95% CI)**	**Odds ratio (95% CI)**	***n***	**% (95% CI)**	**Odds ratio (95% CI)**
**Incident diagnosed diseases**						
Cardiovascular/metabolic disease	22	6.5 (4.3–9.6)	1.68 (1.04–2.71)	177	4.0 (3.5–4.7)	reference
Lung disease	11	3.2 (1.8–5.7)	3.50 (1.73–7.09)	49	1.1 (0.8–1.5)	reference
Gastrointestinal disease	8	2.3 (1.2–4.6)	0.75 (0.36–1.56)	134	3.1 (2.6–3.6)	reference
Liver/kidney disease	9	2.7 (1.4–5.0)	3.70 (1.69–8.08)	39	0.9 (0.7–1.2)	reference
Neurological disease	6	1.8 (0.8–3.9)	1.86 (0.75–4.61)	40	0.9 (0.7–1.2)	reference
Mental disease	9	2.7 (1.4–5.0)	0.93 (0.46–1.91)	140	3.2 (2.7–3.8)	reference
**Subjective measures**						
Current subjective health: fair/bad/very bad	67	19.3 (15.5–23.8)	1.91 (1.39–2.64)	579	13.0 (12.1–14.1)	reference
Subjective memory impairment	89	25.7 (21.4–30.6)	2.27 (1.74–2.98)	633	14.3 (13.3–15.4)	reference

Odds ratio and 95% confidence interval (95% CI) are derived from logistic regression models adjusted for the same variables as in Table 2. Missings for cardiovascular/metabolic disease *n* = 80, lung disease *n* = 65, gastrointestinal disease *n* = 92, liver/kidney disease *n* = 98, neurological disease *n* = 77, mental disease *n* = 130, subjective health *n* = 31, subjective memory impairment *n* = 45

### Subjective health, subjective memory impairment, and health-related quality of life in association with SARS-CoV-2 infection

Participants with SARS-CoV-2 infection at baseline reported a fair, bad or very bad health state at follow-up more frequently than participants without infection (Table 3). Furthermore, participants infected at baseline more frequently reported subjective memory impairment compared to never infected participants. In multivariable regression analyses, SARS-CoV-2 infection status was significantly associated with an about twofold higher odds with impaired subjective health (OR 1.91, 95% CI 1.39–2.64) and subjective memory impairment (2.27, 1.74–2.98). According to estimated PAR, proportions attributable to SARS-CoV-2 infection were 4.9% (95% CI 2.9–6.4%) for worse subjective health and 6.9% (5.2–8.2%) for impaired memory.

Participants with infection significantly differed from participants without SARS-CoV-2 infection in terms of higher mean scores for fatigue and lower mean scores for physical functioning (Table 4). No differences were observed regarding other aspects of health-related quality of life as assessed by the domains of the PROMIS-29 instrument. In multivariable regression analyses, associations between SARS-CoV-2 infection and worse scores for fatigue (β 1.44, 95% CI 0.44–2.43) and physical functioning (-0.92; -1.51- -0.33) remained statistically significant.

**Table 4:** PROMIS domains measuring different aspects of health-related quality of life at follow-up between participants with and without SARS-CoV-2 infection

PROMIS domains	Infection at baseline (*n* = 350)			No infection (*n* = 4467)		
	***n***	**mean score** **(95% CI)**	**β coefficient (95% CI)**	***n***	**mean score** **(95% CI))**	**β coefficient (95% CI)**
Physical functioning ^a^	347	52.7 (52.0–53.5)	-0,92 (-1.51- -0.33)	4434	53.3 (53.2–53.6)	reference
Anxiety symptoms ^b^	347	48.9 (48.0–49.7)	0.37 (-0.45–1.19)	4429	48.6 (48.3–48.8)	reference
Depressive symptoms ^b^	347	49.3 (48.5–50.1)	0.74 (-0.06–1.54)	4430	48.6 (48.4–48.9)	reference
Fatigue ^b^	347	47.4 (46.3–48.4)	1.44 (0.44–2.43)	4430	46.3 (46.0–46.6)	reference
Sleep disturbance ^b^	347	47.5 (46.7–48.4)	0.21 (-0.66–1.08)	4424	47.5 (47.2–47.7)	reference
Ability to participate in social roles/activities ^a^	345	54.2 (53.3–55.2)	0.52 (-0.40–1.43)	4415	53.5 (53.3–53.8)	reference
Pain interference ^b^	346	48.6 (47.7–49.4)	0.19 (-0.61–1.00)	4419	48.5 (48.2–48.7)	reference
Pain intensity ^b^	345	1.87 (1.65–2.10)	0.08 (-0.12–0.28)	4412	1.79 (1.73–1.85)	reference

β coefficient and 95% confidence interval (95% CI) are derived from linear regression models adjusted for the same variables as in Table 2

^a^ Higher scores reflect better outcomes

^b^ Higher scores reflect worse outcomes

## Discussion

In this population-based cohort study with a mean follow-up time of 452 days, we found significant differences in reported health outcomes between participants with laboratory-confirmed or self-reported history of SARS-CoV-2 infection at baseline and those who were not infected at baseline or during follow-up. Accounting for differences in sociodemographic, behavioral and health characteristics at baseline, we observed significant associations between SARS-CoV-2 infection and recurrent or persistent health complaints, such as smell and taste disorders, shortness of breath, pain when breathing, fatigue, weakness in legs, myalgia and joint pain, cough, and subjective memory impairment. Compared to participants without SARS-CoV-2 infection, participants with infection were also more likely to report newly diagnosed diseases of the lung, liver and kidney, and the cardiovascular and metabolic system. Participants with infection significantly more often rated their general health status as fair, bad or very bad than never infected participants. Regarding health-related quality of life domains as assessed by the PROMIS-29 questionnaire, we observed significantly higher fatigue and lower physical functioning scores among participants who were infected compared with those who were not, whereas group differences in depressive and anxiety symptoms, pain, ability for participation in social roles, and sleep disturbance were not evident.

### Comparison with other studies

In the context of a previous SARS-CoV-2 infection, numerous long-term health consequences have been described in the literature. These include a variety of individual symptoms as well as common clusters of symptoms such as fatigue/exhaustion, cognitive impairment, lower respiratory tract symptoms, and smell and taste disorders [12, 13, 27–31]. Most of these symptoms are not long COVID-specific and rather share similarities with symptoms of many other diseases and may affect the daily normal functioning and the quality of life [9, 28, 31–33].

In the present study, we found a significant association of SARS-CoV-2 infection status with seven out of 18 health symptoms known to be frequently persisting or intermittently reoccurring in the post-acute phase of SARS-CoV-2 infection. According to estimated PAR, highest proportions attributable to SARS-CoV-2 infection out of these seven health complaints were smell and taste disorders (17.4%), shortness of breath (11.4%) and pain when breathing (9.6%). This is in line with results from previous population-based cohort studies in various countries as well as from systematic reviews. In an umbrella review, including 23 reviews and 102 primary studies, it was shown that the most frequent symptoms after SARS-CoV-2 infection were breathing difficulties and fatigue, followed by smell and taste disturbances [28]. A large epidemiological study from the Netherlands examined the nature and prevalence of post-COVID-19 condition as part of an ongoing regional cohort study of adults living in the northern part of the country [34]. The authors were hence able to correct for individual symptoms present before COVID-19 and the symptom dynamics in the population without SARS-CoV-2 infection during the pandemic. In 12.7% of patients, symptoms including chest pain, difficulties with breathing, pain when breathing, painful muscles, ageusia or anosmia, tingling extremities, feeling hot and cold alternately, and general tiredness were attributed to COVID-19 [34]. In a large Norwegian cohort study it was observed that altered smell or taste and fatigue were among the symptoms with the highest difference in occurrence (16.6% and 13.6%, respectively) when comparing current symptoms between participants with a COVID-19 diagnosis one year ago and those without COVID-19 [29]. For smell/taste disordes and fatigue, the differences in occurrence between participants with and without baseline SARS-CoV-2 infection were similar in the present study (i.e. 9.4% and 10.8%, respectively, based on observed proportions) and the proportions attributable to SARS-CoV-2 infection (i.e. considering multivariable adjusted odds ratios) were 17.4% and 4.4%, respectively. Further, the association between fatigue and SARS-CoV-2 infection in our study was supported by significantly higher mean fatigue scores as assessed by the PROMIS-29 health-related quality of life questionnaire among participants with baseline infection compared to those never infected.

Cognitive impairment often referred to as “brain fog” was one of the major health complaints reported by patients not recovering from SARS-CoV-2 infection [12, 13, 28]. In line with our results, an association between self-reported cognitive impairment and SARS-CoV-2 infection has been confirmed in previous epidemiological studies [32, 35]. A recent systematic review including only controlled studies applying at least one neuropsychological test emphasized the need for identifying specific domains of cognitive impairment and identified short-term memory and attention to be mainly associated with SARS-CoV-2 infection [36]. While cognitive testing was not possible in the context of the present study, we specifically assessed subjective memory impairment by asking for self-reported memory worsening with associated worries, previously shown to predict the risk of developing cognitive decline and dementia [37, 38]. Nevertheless, this association may be confounded by pre-existing cognitive decline, which we could not consider in multivariable models in the present analysis. 

Cluster analysis of symptoms was beyond the scope of this study due to a limited number of persons with previous SARS-CoV-2 infection. Furthermore, the characterization of PASC phenotypes largely depends on the available data. In lack of a consented list of PASC symptoms, the potential PASC symptoms assessed vary considerably between studies, resulting in the identification of symptom categories, which only partly overlap [12, 29–31, 39]. This highlights the need for harmonized symptom assessment in PASC studies, both regarding the specification of health outcomes and assessment instruments.

Long COVID symptoms may occur solely or in combination and may be associated with impaired quality of life and limitations in daily functioning [28, 33]. Regarding subjective general health, our study showed that participants with baseline infection had a poorer subjective health compared with never infected participants. This seems plausible in the view of the fact that participants with infection also experienced more recurrent and persistent symptoms than never infected participants. The PROMIS-29 Profile is an instrument designed to assess seven domains of patient-reported health-related quality of life [19]. Out of the seven domains, two domains of physical well-being (i.e. fatigue and physical functioning) were found to be significantly different between infected and non-infected participants in our study population, indicating impaired quality of life and limitations in daily functioning. While this is consistent with the higher frequency of reported recurrent or persistent health complaints (including fatigue) and worse subjective general health in our study, we did not find any difference in the mental health domains (i.e. anxiety and depression), although long COVID has been found to be associated with higher levels of psychological distress and perceived stress [31, 40]. It is also noticeable that we observed no difference for the pain inference domain or the pain intensity scale between infected and non-infected participants, in spite of higher reported frequencies of myalgia and joint pain as well as pain when breathing among participants infected at baseline than among non-infected participants. Among potential reasons for these inconsistences are the different time frames covered between the pain measure of PROMIS-29 (last seven days) and the queried health complaints (recurrent or persistent).

Apart from highly complex health symptoms, organ specific complications and new-onset chronic noncommunicable diseases may be possible long-term consequences of SARS-CoV-2 infection, in particular among individuals with a severe course of COVID-19 [10]. An increasing number of controlled studies has also reported significantly higher rates of newly diagnosed chronic health conditions, in adults not necessarily hospitalized for COVID-19 compared with controls, including diseases of the lung, liver, kidney, cardiovascular and metabolic system as well as neurodegenerative diseases and autoimmune diseases [11, 41]. In the present study, lung diseases, liver and kidney diseases as well as cardiovascular and metabolic diseases were more frequently reported as new-onset conditions by participants with infection at baseline than by participants without infection. However, it should be noted that due to the low number of incident cases, results should be interpreted with caution. Nevertheless, another study from Germany published in early 2022 reported significantly more frequent previously unknown multi-organ damage in adults aged 45 to 74 years with predominantly mild disease progression after confirmed SARS-CoV-2 infection compared with a control group [42].

The pathophysiological causes of these observations as well as the underlying mechanisms of health consequences are not yet completely clear and more scientific research efforts are required. However, recent evidence showed that chronic inflammation and occlusion of small vessels (microthrombi) as well as activation of autoimmune processes are involved in the development of long-term health consequences [43–45]. Regarding cognitive impairment after a SARS-CoV-2 infection, potential pathophysiological mechanism have been identified including the activation of the kynurenine pathway [44]. Concerning respiratory conditions after a SARS-CoV-2 infection, an immunological study comparing individuals with long COVID and individuals who had recovered from COVID-19 noted a correlation between decreased lung function, systemic inflammation and SARS-CoV-2-specific T cells [46].

### Strengths and limitations

Given the current challenges in long COVID research that most of the studies are uncontrolled cohorts and include a relatively short follow-up [13], major strengths of this study include the population-based cohort-design, the well-defined groups of SARS-CoV-2-exposed and non-exposed participants, and the relatively long follow-up period of at least 1 year. In addition, we examined multiple potential health consequences of SARS-CoV-2 infection at the follow-up, which included subjective measures of patient-reported health besides commonly investigated symptoms and incident diseases. Further we performed sensitivity analyses, including the application of a stricter definition of the infection status in one municipality and the limitation to recurrent or persistent health complaints that lasted until the date of follow-up, which confirmed the observed differences between infected and non-infected participants from main analysis.

Among the limitations of our study is the uncertainty in the group of never infected participants regarding the occurrence of asymptomatic SARS-CoV-2 infection; however, we could confirm our results when considering the stricter definition of the infection based on follow-up blood samples from one municipality in the sensitivity analysis. In addition, misclassification of infected participants as never infected would result in an attenuation of the observed association. Therefore, the difference between baseline infected and never infected participants might be even greater than calculated in our study. Another limitation of our study is that it was not possible to precisely determine the time interval between the onset of SARS-CoV-2 infection and occurrence of the respective health problem during the follow-up period due to the lack of information on time for the first occurrence of the health problem. Further, all health outcomes of our study were self-reported and no objective tests, e.g. for the measurement of memory impairment, were applied. Also, our study reflects possible long-term health consequences for individuals with a rather mild course of acute SARS-CoV-2 infection and of earlier variants of SARS-CoV-2. When comparing results between studies concerning long COVID, it should be kept in mind that individuals with a severe course of infection or those infected with the later variants, such as omicron, may have a different probability to experience long COVID [47]. Finally, response proportions varied across the four municipalities at baseline and were rather moderate at follow-up, so the representativeness of the study population for the target population at baseline as well as the generalizability of the observed frequencies of symptoms and newly diagnosed diseases at follow-up might be limited.

## Conclusion

Results from this population-based controlled study point to considerable long-term impact of SARS-CoV-2 infection on health and well-being among adults with a mainly mild course of acute infection. Harnessing high-quality population-based epidemiological and health care research on long COVID is necessary to guide the planning and implementation of effective preventive and health care services.

### Supplementary Information

**Additional file 1: Supplementary table 1.** Recurrent or persistent health complaints that are still existing until today comparing participants with and without SARS-CoV-2 infection. **Supplementary figure 1.** Number of recurrent or persistent health complaints that are still existing until today comparing participants with and without SARS-CoV-2. **Supplementary table 2.** Recurrent or persistent health complaints comparing participants with and without SARS-CoV-2 infection applying an extended definition of SARS-CoV-2 infection in one municipality.

## Data Availability

The dataset analyzed is not publicly available due to ethical reasons. The corresponding author may be contacted for more information.

## Abbreviations

BMI: Body mass index
CI: Confidence interval
CoMoLo: CORONA-MONITORING lokal
COVID-19: Coronavirus disease 2019
IgG: Immunoglobulin
PASC: Post-acute sequelae of SARS-CoV-2 infection
PAR: Population-attributable risk
PCR: Polymerase chain reaction
PROMIS: Patient-Reported Outcomes Measurement Information System
SARS-CoV-2: Severe acute respiratory syndrome coronavirus type 2
OR: Odds ratio
