# Supplementary material for: Long-term health consequences among individuals with SARS-CoV-2 infection compared to individuals without infection: results of the population-based cohort study CoMoLo Follow-up
Source: BMC Public Health. 2023 Aug 21;23:1587. doi: 10.1186/s12889-023-16524-8 (PMC10440884; doi:10.1186/s12889-023-16524-8)
Supplement: Supplementary file 1 — Additional file 1: Supplementary table 1. Recurrent or persistent health complaints that are still existing until today comparing participants with and without SARS-CoV-2 infection. Supplementary figure 1. Number of recurrent or persistent health complaints that are still existing until today comparing participants with and without SARS-CoV-2. Supplementary table 2. Recurrent or persistent health complaints comparing participants with and without SARS-CoV-2 infection applying an extended definition of SARS-CoV-2 infection in one municipality. [file 12889_2023_16524_MOESM1_ESM.pdf]

Supplementary table 1 Recurrent or persistent health complaints *that are still existing until today* comparing participants with and without SARS-CoV-2 infection

**Recurrent or persistent health complaints still existing until today**	**Infection at baseline (*n*=350)**			**No infection (*n*=4467)**		
	n	**Proportion in % (95% CI)**	**Odds ratio (95% CI)**	***n***	**Proportion in % (95% CI)**	**Odds ratio (95% CI)**
Fever above 38 °C	2	0.6 (0.1-2.3)	2.78 (0.55-13.95)	8	0.2 (0.1-0.4)	reference
Cough	31	9.0 (6.4-12.5)	1.51 (1.01-2.25)	280	6.5 (5.8-7.3)	reference
Shortness of breath	63	18.3 (14.6-22.8)	4.16 (2.95-5.86)	282	6.5 (5.8-7.3)	reference
Pain when breathing	7	2.0 (1.0-4.2)	4.49 (1.77-11.36)	21	0.5 (0.3-0.7)	reference
Chest pain	14	4.1 (2.4-6.8)	1.85 (1.01-3.39)	99	2.3 (1.9-2.8)	reference
Sore throat	12	3.5 (2.0-6.1)	1.10 (0.59-2.02)	135	3.1 (2.6-3.7)	reference
Runny nose	30	8.7 (6.2-12.2)	1.19 (0.80-1.78)	334	7.7 (7.0-8.6)	reference
Smell/taste disorders	25	7.3 (5.0-10.5)	4.77 (2.91-7.82)	70	1.6 (1.3-2.0)	reference
Loss of appetite	9	2.6 (1.4-5.0)	2.08 (0.98-4.42)	63	1.5 (1.1-1.9)	reference
Fatigue	86	25.0 (20.7-29.9)	2.12 (1.60-2.81)	644	14.9 (13.9-16.0)	reference
Headache	40	11.7 (8.7-15.5)	1.15 (0.81-1.65)	442	10.2 (9.4-11.2)	reference
Dizziness	33	9.6 (6.9-13.2)	1.37 (0.91-2.07)	314	7.3 (6.5-8.1)	reference
Nausea/stomach upset	15	4.4 (2.6-7.1)	1.12 (0.63-1.98)	169	3.9 (3.4-4.5)	reference
Hot flushes/chills	22	6.4 (4.2-9.5)	0.96 (0.60-1.54)	278	6.4 (5.7-7.2)	reference
Sleep disorders	61	17.7 (14.0-22.2)	1.06 (0.77-1.45)	736	17.0 (16.0-18.2)	reference
Myalgia and joint pain	44	12.8 (9.7-16.8)	1.41 (0.97-2.04)	392	9.1 (8.3-10.0)	reference
Numbness/burning/tingling in the feet/legs/hands	22	6.4 (4.3-9.6)	1.20 (0.73-1.97)	236	5.5 (4.8-6.2)	reference
Weakness in the legs	35	10.2 (7.4-13.9)	2.00 (1.30-3.06)	255	5.9 (5.2-6.6)	reference

Odds ratio and 95% confidence interval (95% CI) are derived from logistic regression models adjusted for baseline age (years), sex, municipality, baseline educational level (lower, medium, high), baseline body mass index (≥30 vs. <30 kg/m^2), baseline smoking status (current, ex-, never smoker), chronic disease or health problem lasting for at least 6 months before baseline (yes vs. no), health-related limitation in usual everyday activities lasting for at least 6 months before baseline (yes vs. no), depression/anxiety symptoms in the past two weeks before baseline (yes vs. no) and follow-up time (days). Missings for loss of appetite, dizziness (each n=149), shortness of breath, nausea/stomach upset, hot flushes/chill, weakness in the legs, numbness/burning/tingling in the feet/legs/hands (each n=151), fever above 38 °C, cough, pain when breathing, sore throat (each n=152), smell/taste disorders, fatigue, headache (each n=153), runny nose (n=154), sleep disorders, myalgia and joint pain (each n=155), chest pain (n=158).

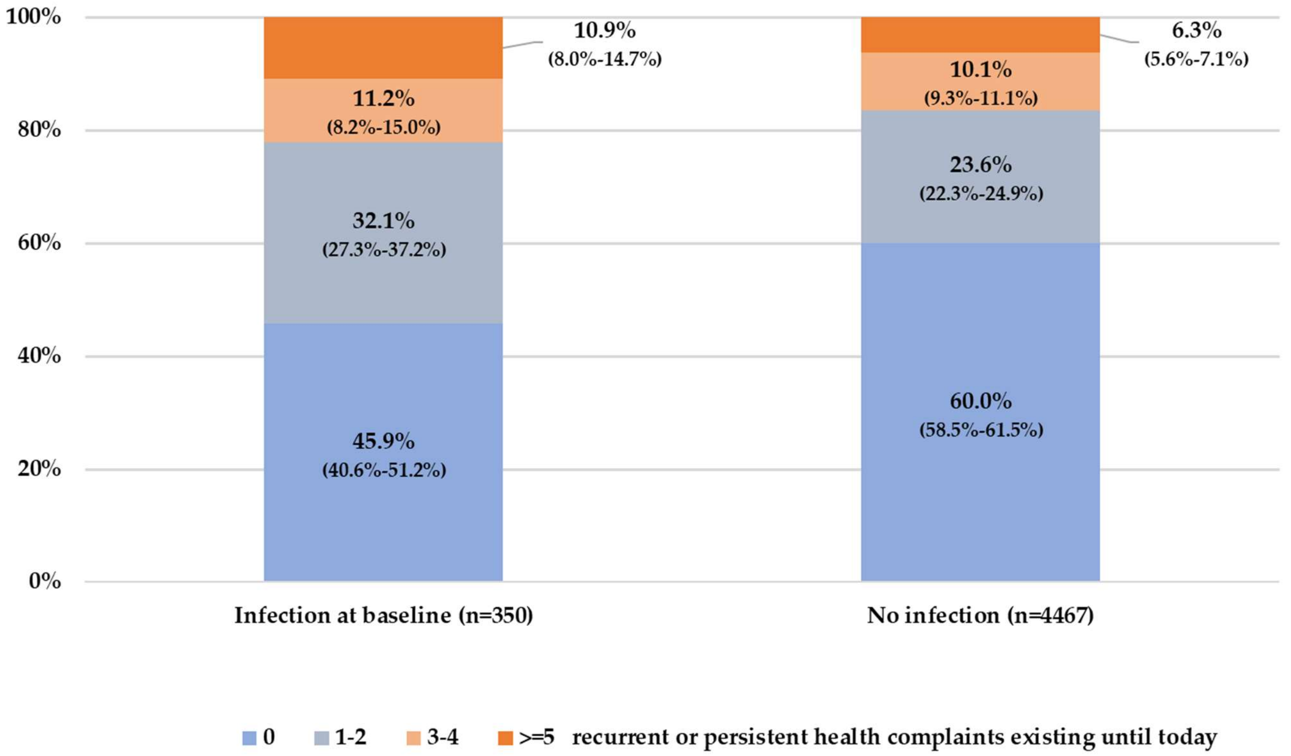

Supplementary figure 1 Number of recurrent or persistent health complaints *that are still existing until today* comparing participants with and without SARS-CoV-2

Supplementary table 2 Recurrent or persistent health complaints comparing participants with and without SARS-CoV-2 infection *applying an extended definition of SARS-CoV-2 infection in one municipality*

Recurrent or persistent health complaints still existing until today	Infection at baseline (*n*=42)			No infection (*n*=1256)		
	n	Proportion in % (95% CI)	Odds ratio (95% CI)	*n*	Proportion in % (95% CI)	Odds ratio (95% CI)
Fever above 38 °C	3	7.1 (2.2-20.5)	1.66 (0.47-5.79)	49	4.1 (3.1-5.4)	reference
Cough	13	31.0 (18.6-46.8)	1.82 (0.91-3.64)	244	20.5 (18.3-22.9)	reference
Shortness of breath	14	33.3 (20.5 -49.2)	5.90 (2.82-12.34)	135	11.3 (9.6-13.3)	reference
Pain when breathing	1	2.4 (0.3-15.8)	1.33 (0.16-10.92)	20	1.7 (1.1-2.6)	reference
Chest pain	3	7.1 (2.2-20.5)	1.91 (0.55-6.57)	59	5.0 (3.9-6.3)	reference
Sore throat	11	26.2 (14.9-41.9)	1.44 (0.69-3.02)	244	20.5 (18.3-22.9)	reference
Runny nose	15	35.7 (22.5-51.6)	1.24 (0.64-2.42)	373	31.3 (28.7-34.0)	reference
Smell/taste disorders	7	16.7 (8.0-31.6)	6.06 (2.46-14.92)	37	3.1 (2.3-4.3)	reference
Loss of appetite	1	2.4 (0.3-15.8)	0.67 (0.09-5.21)	52	4.4 (3.3-5.7)	reference
Fatigue	14	33.3 (20.5-49.2)	1.97 (0.98-3.95)	278	23.3 (21.0-25.8)	reference
Headache	13	31.0 (18.6-46.8)	1.01 (0.49-2.08)	376	31.5 (29.0-34.2)	reference
Dizziness	6	14.3 (6.4-28.9)	1.01 (0.41-2.53)	185	15.5 (13.6-17.7)	reference
Nausea/stomach upset	5	11.9 (4.9-26.1)	1.17 (0.44-3.11)	140	11.7 (10.0-13.7)	reference
Hot flushes/chills	6	14.3 (6.4-28.9)	1.34 (0.53-3.38)	135	11.3 (9.6-13.3)	reference
Sleep disorders	11	26.2 (14.9-41.9)	1.33 (0.64-2.79)	280	23.5 (21.2-26.0)	reference
Myalgia and joint pain	7	16.7 (8.0-31.6)	1.51 (0.62-3.71)	165	13.8 (12.0-15.9)	reference
Numbness/burning/tingling in the feet/legs/hands	1	2.4 (0.3-15.8)	0.33 (0.04-2.64)	96	8.1 (6.6-9.7)	reference
Weakness in the legs	5	11.9 (4.9-26.1)	1.76 (0.63-4.89)	105	8.8 (7.3-10.6)	reference

Odds ratio and 95% confidence interval (95% CI) are derived from logistic regression models adjusted for the same variables as in Supplementary table 1. Missings for fever above 38 °C, cough, shortness of breath, sore throat, runny nose, loss of appetite, fatigue, headache, dizziness, nausea/stomach upset, hot flushes/chills, sleep disorders, myalgia and joint pain, numbness/burning/tingling in the feet/legs/hands, pain when breathing (each n=64), chest pain, smell/taste disorders, weakness in the legs (each n=65).
